# Vanillin Promotes Osteoblast Differentiation, Mineral Apposition, and Antioxidant Effects in Pre-Osteoblasts

**DOI:** 10.3390/pharmaceutics16040485

**Published:** 2024-04-01

**Authors:** Hyung-Mun Yun, Eonmi Kim, Yoon-Ju Kwon, Kyung-Ran Park

**Affiliations:** 1 Department of Oral and Maxillofacial Pathology, School of Dentistry, Kyung Hee University, Seoul 02447, Republic of Korea; 2 National Development Institute of Korean Medicine, Gyeongsan 38540, Republic of Korea; minnie60@nikom.or.kr (E.K.); iamsook@nikom.or.kr (Y.-J.K.); 3 Korea Basic Science Institute (KBSI), Gwangju 61751, Republic of Korea

**Keywords:** BMP2, bone, Cbfa1, mineralization, osteoblast, osteogenesis, ROS, RUNX2, vanillin

## Abstract

Antioxidant vanillin (4-hydroxy-3-methoxybenzaldehyde) is used as a flavoring in foods, beverages, and pharmaceuticals. Vanillin possesses various biological effects, such as antioxidant, anti-inflammatory, antibacterial, and anticancer properties. This study aimed to investigate the biological activities of vanillin purified from *Adenophora triphylla var. japonica* Hara on bone-forming processes. Vanillin treatment induced mineralization as a marker for mature osteoblasts, after stimulating alkaline phosphatase (ALP) staining and activity. The bone-forming processes of vanillin are mainly mediated by the upregulation of the bone morphogenetic protein 2 (BMP2), phospho-Smad1/5/8, and runt-related transcription factor 2 (RUNX2) pathway during the differentiation of osteogenic cells. Moreover, vanillin promoted osteoblast-mediated bone-forming phenotypes by inducing migration and F-actin polymerization. Furthermore, we validated that vanillin-mediated bone-forming processes were attenuated by noggin and DKK1. Finally, we demonstrated that vanillin-mediated antioxidant effects prevent the death of osteoblasts during bone-forming processes. Overall, vanillin has bone-forming properties through the BMP2-mediated biological mechanism, indicating it as a bone-protective compound for bone health and bone diseases such as periodontitis and osteoporosis.

## 1. Introduction

Osteoblasts are most active during embryonic bone development, but osteoblasts are also activated when a defect needs to be repaired or the bone matrix is regenerated throughout life. Osteoblasts are generated from osteogenic cells such as mesenchymal stem cells, and the osteoblast-mediated bone-forming function is important for skeletal health. Once the osteoblast master regulator, runt-related transcription factor 2 (RUNX2), is activated, the osteogenic cells exit the cell cycle and begin to differentiate into osteoblasts, and their maturation forms the bone matrix mineralization with proteins such as alkaline phosphatase (ALP), collagen, and osteocalcin. The RUNX2 upstream osteogenic factors involving bone morphogenetic protein 2 (BMP2) and Wnt3a are the most researched. The osteogenic factors stimulate osteoblast differentiation, maturation, and bone formation [1]. However, abnormal osteoblast regulation and death can cause insufficient or excessive bone formation, which can lead to the onset of serious bone diseases such as periodontitis and osteoporosis [1,2,3]. The identification of new molecular targets in the mechanisms regulating the patho/physiological osteogenic processes could provide a valuable therapeutic understanding to prevent or treat bone diseases. Clinically, this study using potential compounds known to be relatively safe and suitable for long-term treatment is also critical for the health of the skeletal system to prevent bone diseases such as osteoporosis.

With the growing interest in health, plants are employed as natural resources, including functional foods, cosmetics, pharmaceuticals, and raw materials for high-value industries [4,5]. *Adenophora triphylla var. japonica* Hara (also known as Shasham or Jan-dae in Korea) in the family Campanulaceae is an erect perennial herb that grows up to 100 cm and grows well in warm, sunny locations or slightly shaded crevices [4]. *A. triphylla* is commonly utilized as an oriental medicinal herb in Korea, China, and Japan for ailments such as bronchitis, cancer, cough, inflammation, and obesity [4,6]. Originally, it was noted as a remedy for lung fever, old coughs, and somnolence in China in 1578, and in Korea in 1613 as a herbal medicine that energizes the lungs [7]. It contains bioactive compounds such as alkaloids, inulin, piperidine, lupenone, saponin, triphyllol, and triterpenoids, which exhibit antibacterial, anticancer, antidiabetic, anti-inflammatory, antioxidant, detoxification, mucus-producing, hepatoprotective, and neuroprotective effects [4,7,8,9,10,11,12,13,14]. In cell differentiation effects, a polyphenolic compound lupenone from *A. triphylla* was reported to suppress adipocyte differentiation through the downregulation of Peroxisome proliferator-activated receptor γ and CCAAT-enhancer-binding protein α in 3T3-L1 cells [13]. However, the biological effects of *A. triphylla* extracts on osteoblast differentiation were not investigated.

A phenolic compound known as vanillin (4-hydroxy-3-methoxybenzaldehyde) is commonly used in food and drink, including milk powder, candies, and biscuits [15]. Due to its stability, low toxicity, pleasant aroma, and antibacterial function, vanillin has also been frequently utilized as an ingredient and flavoring in foods, drinks, and cosmetics [16]. Vanillin is safe and accessible to all; therefore, the health benefits of vanillin for humans are worthy of attention [17]. It was suggested that the beneficial effects of vanillin might be more advantageous for daily health care, and these vanillin-rich diets can decrease free radicals that cause tumorigenesis [18,19]. In the skeletal system, it was reported that vanillin has an inhibitory effect on osteoclast differentiation through mitochondrial-dependent apoptosis in RAW264.7 cells [20].

To date, the biological effects of vanillin on osteoblasts have not been investigated. Therefore, it is worth exploring the possible function and biological mechanism of vanillin on osteoblast differentiation, maturation, and survival for the health of the skeletal system. This study aimed to investigate the bone-protective roles of antioxidant vanillin isolated (>99.99% purity) from *A. triphylla* in the biological mechanisms regulating the osteogenic processes, using calvaria-derived osteogenic MC3T3-E1 cells as an in vitro cell system.

## 2. Materials and Methods

### 2.1. Plant Material and Isolation of Vanillin from Adenophora triphylla var. japonica Hara

The *Adenophora triphylla var. japonica* Hara was purchased at the commercial herbal medicine market. A voucher specimen (P392) from *A. triphylla* has been deposited in the Natural Products Bank, National Institute for Korean Medicine Development (NIKOM, Gyeongsan-si, Republic of Korea). ^1^H NMR and ^13^C NMR spectra were obtained using a JEOL ECX-500 spectrometer (JEOL Ltd., Tokyo, Japan). High-performance liquid chromatography (HPLC) was performed on the Agilent 1260 series (Agilent Inc., Palo Alto, CA, USA) with a quaternary pump, diode array detector (DAD), and evaporative light-scattering detector (ELSD), together with a C18 column (YMC Pack Pro C18, 5 μm, 250 mm × 4.6 mm) (YMC Co., Ltd., Kyoto, Japan). Column chromatography was conducted using Silica gel 60 (70–230 mesh ASTM, Merck, Darmstadt, Germany), ODS-A (YMC Co., Ltd., Kyoto, Japan), and sephadex LH-20 (GE Healthcare, Chicago, IL, USA).

The *A. triphylla var. japonica* Hara (4.6 kg) was extracted over 2 days with MeOH at room temperature. The crude extract (990.0 g) was suspended in distilled water (DW) and then solvent-partitioned with *n*-butanol (BuOH). The BuOH soluble fractions (33.2 g) were divided with normal-phase silica gel VLC using a step gradient mixture of CH_2_Cl_2_, MeOH, and water (40:10:1 → 70:30:3 → 60:40:4, *v*/*v*) to yield 28 fractions. Fr. 19 (2.1 g) was subjected to reverse-phase (ODS-A) column chromatography eluted with a gradient system of MeOH-H_2_O (70:30 to 30:70, *v*/*v*) to obtain 3 fractions. Fr.19-2 (250.0 mg) was applied to sephadex LH-20 column chromatography with isocratic solvent condition (MeOH/H_2_O = 70:30, *v*/*v*) to obtain an active single compound, vanillin (white crystalline, 80.0 mg).

### 2.2. Vanillin Stock Solution

Vanillin stock solution (1000×) was dissolved in 100% dimethyl sulfoxide (DMSO) (Sigma-Aldrich, St. Louis, MO, USA), and 0.1% DMSO was included in the working solution to ensure no toxicity to cells. As the vehicle control, 0.1% DMSO was used.

### 2.3. Osteogenic Cells and Osteoblast Differentiation

Osteogenic MC3T3E-1 cells (#CRL-2593) were obtained from the American Type Culture Collection (Manassas, VA, USA). The cells were cultured in an atmosphere of 37 °C, 5% CO_2_, and 95% air with 10% Gibco FBS (Thermo Fisher Scientific, Waltham, MA, USA) and 1× Gibco antibiotic–antimycotic (Thermo Fisher Scientific) in α-minimum essential medium (α-MEM) without l-ascorbic acid (L-AA) (WELGEME, Inc., Seoul, Republic of Korea).

Osteoblast differentiation was induced using osteogenic supplement medium (OS) containing 50 µg/mL L-AA (Sigma-Aldrich) and 10 mM β-glycerophosphate (β-GP) (Sigma-Aldrich), with or without vanillin. During osteoblast differentiation, the OS was changed at 2 days.

### 2.4. Cell Toxicity Analysis

A 3-[4,5-dimethylthiazol-2-yl]-2,5-diphenyltetrazolium bromide (MTT) assay (Sigma-Aldrich) was used to analyze cell toxicity in the osteogenic cells, as previously described [3]. Briefly, vanillin was incubated with osteogenic cells for 24 h, and then the cells were treated with 20 µL of 1000× MTT solution (5 mg/mL in PBS) for 4 h; the crystalline dark purple product, formazan, was solubilized in 100% DMSO (Sigma-Aldrich), and the absorbance was measured at a wavelength of 540 nm using a Multiskan GO Microplate Spectrophotometer (Thermo Fisher Scientific).

### 2.5. Early Osteoblast Differentiation Analysis

Early osteoblast differentiation was performed as previously described [21]. Briefly, the osteoblast differentiation was induced in OS with vanillin, and alkaline phosphatase (ALP) staining (Takara Bio Inc., Shiga, Japan) and activity (Biovision, Milpitas, CA, USA) assays were measured after 7 days, in accordance with the manufacturer’s procedure. The absorbance of ALP activity was measured at a wavelength of 405 nm using a Multiskan GO Microplate Spectrophotometer (Thermo Fisher Scientific).

### 2.6. Late/Terminal Osteoblast Differentiation Analysis

An Alizarin red S (ARS) staining assay was used to analyze late osteoblast differentiation as previously described [3]. Briefly, osteoblast differentiation was induced in OS with vanillin for 14 days. Subsequently, the cells were stained for 15 min using a 2% ARS solution (pH 4.2) from Sigma-Aldrich. The staining level was measured at a wavelength of 590 nm using a Multiskan GO Microplate Spectrophotometer (Thermo Fisher Scientific).

### 2.7. Western Blot Analysis

Western blot analysis was performed as previously described [3]. Briefly, cells were treated in OS with vanillin for 3 days and then lysed using a lysis buffer. The total protein concentration was determined using Bradford reagent (Bio-Rad, Hercules, CA, USA), and the equal proteins were transferred to polyvinylidene fluoride membranes (Millipore, Bedford, MA, USA). The membranes were incubated with blocking solution (5% skim milk in 1×TBS containing 0.05% Tween-20 (TBST)) at room temperature for 1 h and incubated with primary antibodies at 4 °C overnight. The membranes were washed with 1×TBST and incubated with horseradish peroxidase–secondary antibodies (1:10,000; Jackson ImmunoResearch, West Grove, PA, USA) at room temperature for 1 h. Protein signals were detected using the ProteinSimple detection system (ProteinSimple Inc., Santa Clara, CA, USA).

The following antibodies were used: AKT (1:1000, #4691, Cell Signaling Technology, Beverly, MA, USA), p-AKT (1:1000, #4060, Cell Signaling Technology), β-actin (1:1000, #sc-47778, Santa Cruz Biotechnology, Santa Cruz, CA, USA), p-ERK1/2 (1:1000, #9101S, Cell Signaling Technology), ERK1/2 (1:2000, #9102, Cell Signaling Technology), p-JNK (1:1000, #9251, Cell Signaling Technology), JNK (1:1000, #9252, Cell Signaling Technology), p-p38 (1:1000, #9211, Cell Signaling Technology), p38 (1:1000, #9212, Cell Signaling Technology), RUNX2 (1:1000, #12556, Cell Signaling Technology), BMP2 (1: 500, #CSB-PAO9419AORb; CUSABIO, Houston, TX, USA), p-GSK3β (1:1000, #9336, Cell Signaling Technology), GSK3β (1:1000, #12456, Cell Signaling Technology), p-Smad1/5/8 (1:2000, #13820, Cell Signaling Technology), and Wnt3a (1:1000, #2721; Cell Signaling Technology).

### 2.8. Immunocytochemistry Analysis

Immunocytochemistry was performed as previously described [22]. Briefly, cells were treated in OS with vanillin for 3 days. Subsequently, they were fixed using a 4% formalin solution, permeabilized with a 0.1% Triton X-100 solution, and blocked with a 5% BSA blocking solution at room temperature. RUNX2 was immunostained using anti-RUNX2 antibody (1:200; Cell Signaling Technology, Beverly, MA, USA) and Alexa-Fluor 488-conjugated secondary antibodies (1:400; Invitrogen, Carlsbad, CA, USA). Nuclei were stained with PI solution (Sigma-Aldrich), and the slides (Thermo Fisher Scientific) were mounted with Fluoromount Aqueous Mounting Medium (Sigma-Aldrich). Immunofluorescence signals against RUNX2 and PI were captured using intravital multi-photon microscope system (IMPM) microscopy in the Korea Basic Science Institute (KBSI, Daejeon, Republic of Korea).

### 2.9. F-Actin Polymerization Analysis

A phalloidin and DRAQ5 staining assay was used to analyze F-actin polymerization. Osteoblasts were fixed using a 4% formalin solution, permeabilized with a 0.1% Triton X-100 solution, and stained using phalloidin (Thermo Fisher Scientific) at room temperature for 30 min. Nuclei were stained with a DRAQ5 solution (Thermo Fisher Scientific), and the slides (Thermo Fisher Scientific) were mounted using Fluoromount Aqueous Mounting Medium (Sigma-Aldrich). Immunofluorescence signals against RUNX2 and PI were captured using IMPM microscopy in the Korea Basic Science Institute (KBSI).

### 2.10. Cell Migration Analysis

The cell migration assay was analyzed using a Boyden chamber with membranes coated with Matrigel solution (Corning Life Sciences, Tewksbury, MA, USA) as previously described [22]. Briefly, a Nuclepore filter was coated with Matrigel. The Boyden chamber was used for 4 h of incubation, before the cells were fixed using a 4% formalin solution and then stained with a 0.5% crystal violet solution. The migrated osteoblasts were detected using a light microscope.

### 2.11. ROS and Active Mitochondria Staining Analyses

ROS levels were detected using CellROX™ Green reagent (Invitrogen, Carlsbad, CA, USA), and active mitochondria were detected using MitoTracker™ Red CMXRos (Invitrogen). Osteoblasts were incubated in an atmosphere at 37 °C with 5% CO_2_ and 95% air, using either 5 μM CellROX™ Green reagent or 500 nM MitoTracker™ Red CMXRos for 30 min. The cells were fixed using a 4% formalin solution, and the nuclei were stained with a 1 mg/mL DAPI solution (Sigma-Aldrich). The slides (Thermo Fisher Scientific) were mounted with Fluoromount Aqueous Mounting Medium (Sigma-Aldrich), and the images were captured using an Olympus IX73 inverted microscope (Olympus Corporation, Tokyo, Japan).

### 2.12. Noggin and DKK1 Inhibitors

Cells were pretreated with a BMP2 inhibitor, 10 μg/mL noggin (PeproTech, Cranbury, NJ, USA), or a Wnt3a inhibitor, 0.5 μg/mL DKK1 (PeproTech, NJ, USA) for 1 h, and then were treated with OS with vanillin for 7 days and 14 days.

### 2.13. Statistical Analysis

The data were analyzed using the GraphPad Prism version 5 program (GraphPad Software, Inc., San Diego, CA, USA). Data are presented as mean ± standard deviation (SD). Statistical significance (*p* < 0.05) was performed on the data using one-way analysis of variance with Dunnett’s post hoc test.

## 3. Results

### 3.1. Extraction and Characterization of Vanillin from the Adenophora triphylla var. japonica Hara

**Figure 1:**
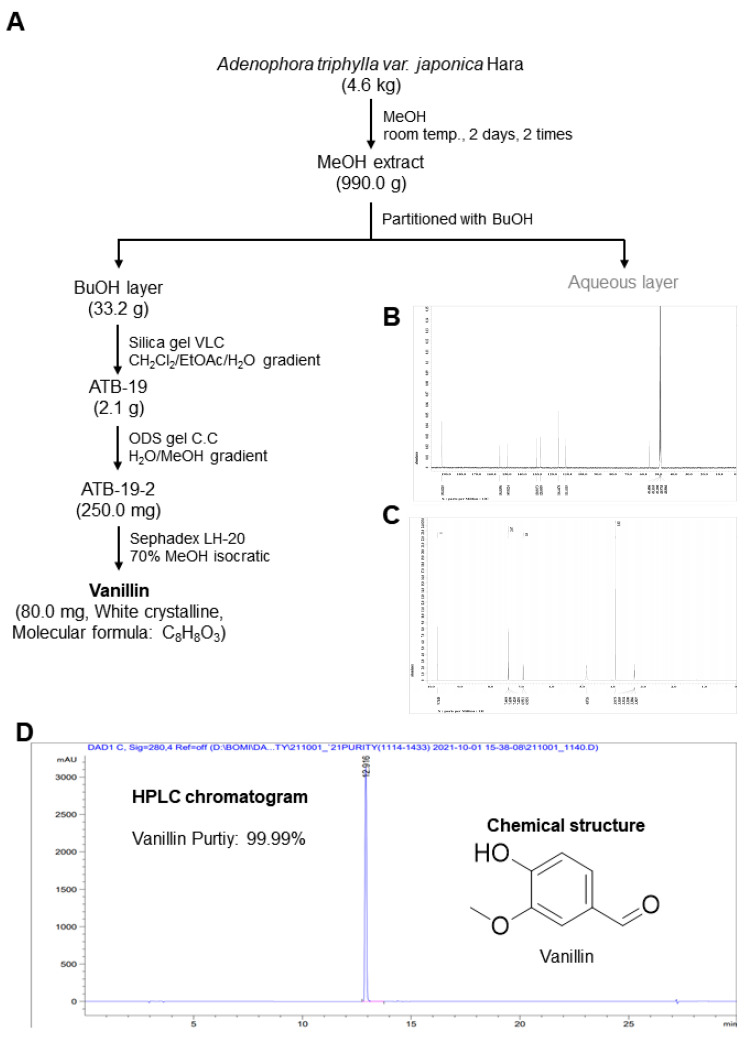
Purification procedure, characterization, and structure of vanillin isolated from *Adenophora triphylla var. japonica* Hara. (**A**) Strategy for the isolation of vanillin. (**B**,**C**) ^13^C NMR (100 MHz, CD_3_OD) (**B**) and ^1^H NMR (400 MHz, CD_3_OD) (**C**) spectra obtained using JEOL ECX-500 spectrometer. (**D**) HPLC and chemical structure of vanillin (C_8_H_8_O_3_, Purity: >99.99%).

Antioxidant vanillin was isolated from the MeOH extract of *Adenophora triphylla var. japonica* Hara (4.6 kg) following a purification procedure (Figure 1A). The vanillin was molecularly characterized using nuclear magnetic resonance (NMR) as follows: molecular formula C_8_H_8_O_3_, ^1^H-NMR (400 MHz, CD_3_OD) δ 9.75 (1H, s -CHO), 7.43 (1H, dd, *J* = 8.0, 1.8 Hz, H-6), 7.35 (1H, d, *J* = 1.8 Hz, H-2), 6.94 (1H, d, *J* = 8.0 Hz, H-5), 3.88 (1H, s, -OCH_3_) (Figure 1B).; ^13^C-NMR (100 MHz, CD_3_OD) δ 191.6 (C-1′), 153.4 (C-3), 148.4 (C-2), 129.4 (C-1), 126.6 (C-5), 115.0 (C-4), 110.0 (C-2), 55.0 (C-3′) (Figure 1C). The chemical structure and high-performance liquid chromatography (HPLC) results (molecular formula: C_8_H_8_O_3_, purity > 99.99%) are displayed in Figure 1D.

### 3.2. Vanillin Promotes Early and Late Osteoblast Differentiation without Cytotoxicity in Osteogenic Cells

**Figure 2:**
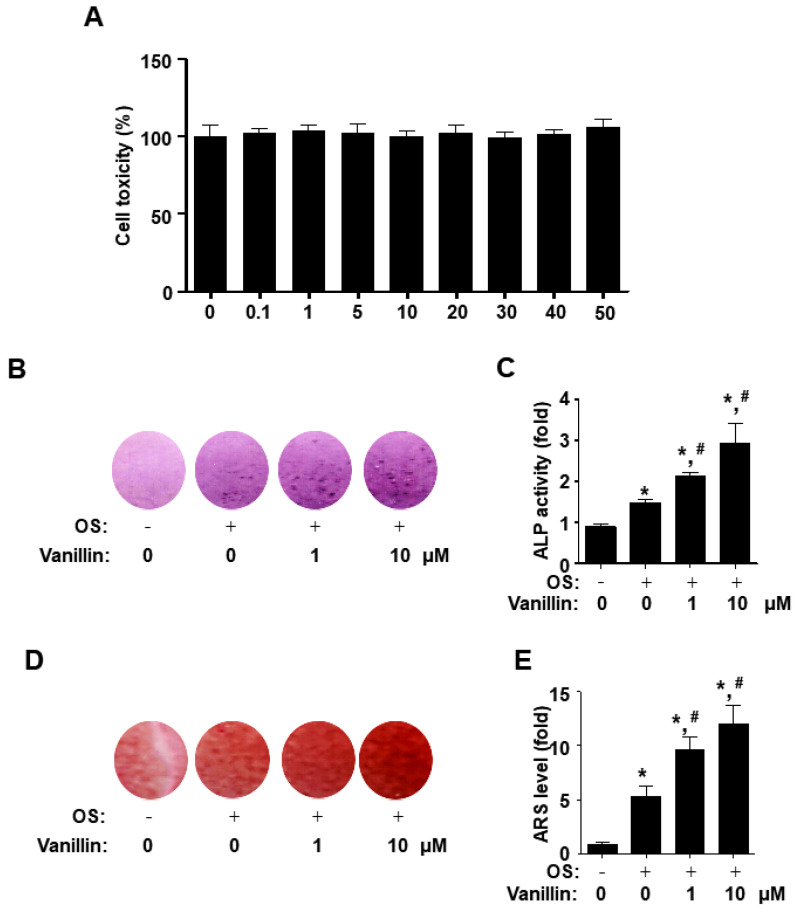
Effects of vanillin on cytotoxicity and osteoblast differentiation in osteogenic cells. (**A**) Cell toxicity was determined in osteogenic cells using the MTT assay. (**B**–**E**) Cells were treated in osteogenic supplement medium (OS) containing 50 μg/mL L-ascorbic acid and 10 mM β-glycerophosphate with vanillin for 7 days (**B**,**C**) and 14 days (**D**,**E**). Osteoblast differentiation was analyzed by ALP staining (**B**), ALP activity (**C**), and ARS staining (**D**). ARS stains were eluted, and the ARS staining level was measured at 590 nm (**E**). Data are expressed as the mean ± SD. * *p* < 0.05, versus control. # *p* < 0.05, versus OS. Data represent the results of three experiments.

To explore the cell toxicity of the characterized vanillin, we treated osteogenic cells with 0.1–50 μM vanillin and analyzed the subsequent cell viability using an MTT assay. The results indicated that vanillin shows no evident cytotoxic effects on osteogenic cells compared with the control (Figure 2A).

The alkaline phosphatase (ALP) enzyme level is a key marker in the early osteoblast differentiation of osteogenic cells. Next, to determine the osteogenic effects of vanillin, we monitored the staining and activity of ALP. Amounts of 1 and 10 μM of vanillin were treated in osteogenic supplement medium (OS) for 7 days, and the osteogenic activities were monitored using ALP staining and activity assays. The observation revealed that vanillin-treated cells increased their ALP staining levels, compared with the cells incubated with control and OS alone (Figure 2B). Consistently, our data showed that vanillin statistically stimulated ALP activity, compared with the cells incubated with control and OS alone (Figure 2C). Subsequently, we placed 1 and 10 μM of vanillin in osteogenic cells for 14 days and determined terminal osteoblast differentiation and maturation by monitoring bone matrix mineralization, using ARS staining to evaluate calcium deposits. The observation showed that vanillin statistically increased ARS staining levels compared with cells incubated with control and OS alone (Figure 2D,E). Thus, these data indicated that vanillin promotes osteogenic processes and induces osteoblast maturation.

### 3.3. Vanillin Enhances Cbfa1 (RUNX2) Expression via the BMP2-Samd1/5/8 Pathway in Osteogenic Cells

**Figure 3:**
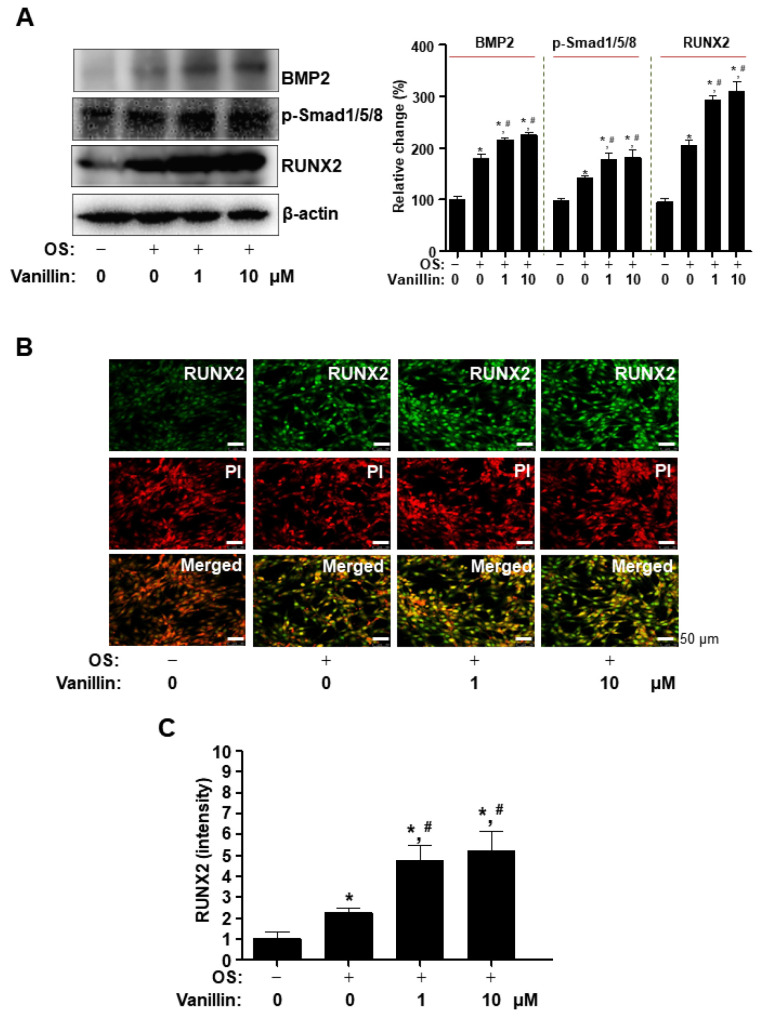
Effects of vanillin on the activation of BMP2 signaling in osteoblasts. (**A**–**C**) Cells were treated in OS with vanillin for 3 days. Western blot analysis was performed to investigate the expression of BMP2, p-Smad1/5/8, RUNX2, and β-actin (**A**). RUNX2 was immunostained with rabbit anti-RUNX2 antibody, followed by Alex488-conjugated secondary antibody (green). And then, the cells were stained with PI (red). Images shown in the upper and middle panels were observed using multiphoton microscopy, and the images are merged in the lower bottom panels (**B**). The relative intensity is shown as a bar graph (**C**). Scale bar: 50 μm. Data are expressed as the mean ± SD. * *p* < 0.05, versus control. # *p* < 0.05, versus OS. Data represent the results of three experiments.

Bone morphogenetic protein 2 (BMP2) is clinically used as an ectopic bone inducer in multi-functional growth factors belonging to the transforming growth factor-beta superfamily. BMP2 regulates the osteoblast differentiation of osteogenic cells through its intracellular signaling proteins. To investigate the action of mechanisms on osteogenic-promoting effects by vanillin in osteogenic cells, we examined the main osteogenic BMP2-Samd1/5/8 pathway. Amounts of 1 and 10 μM of vanillin were treated in osteogenic supplement medium (OS), and the main target molecules were detected using Western blot analysis. The data showed that, compared with the cells incubated with control and OS alone, vanillin-treated cells increased BMP2 expression and its downstream signaling protein, Smad1/5/8, phosphorylation, as well as the expression of a key target gene, Cbfa1 (RUNX2), which is a master transcription factor for osteoblast differentiation (Figure 3A). To validate RUNX2 expression in the nucleus, we observed the cellular localization of RUNX2 using an immunofluorescence assay with DAPI (a nuclear marker). The fluorescence data demonstrated that vanillin statistically increased nuclear RUNX2 expression, compared with the cells incubated with control (10 μM: *p* value = 0.0138) and OS alone (10 μM: *p* value = 0.0378) (Figure 3B). Thus, these data suggest that the osteogenic-promoting effects of vanillin are related to RUNX2 through the BMP2-Samd1/5/8 pathway.

### 3.4. Vanillin Partially Enhances Wnt3 Signaling, Which Is Closely Associated with BMP2 Signaling in Osteogenic Cells

**Figure 4:**
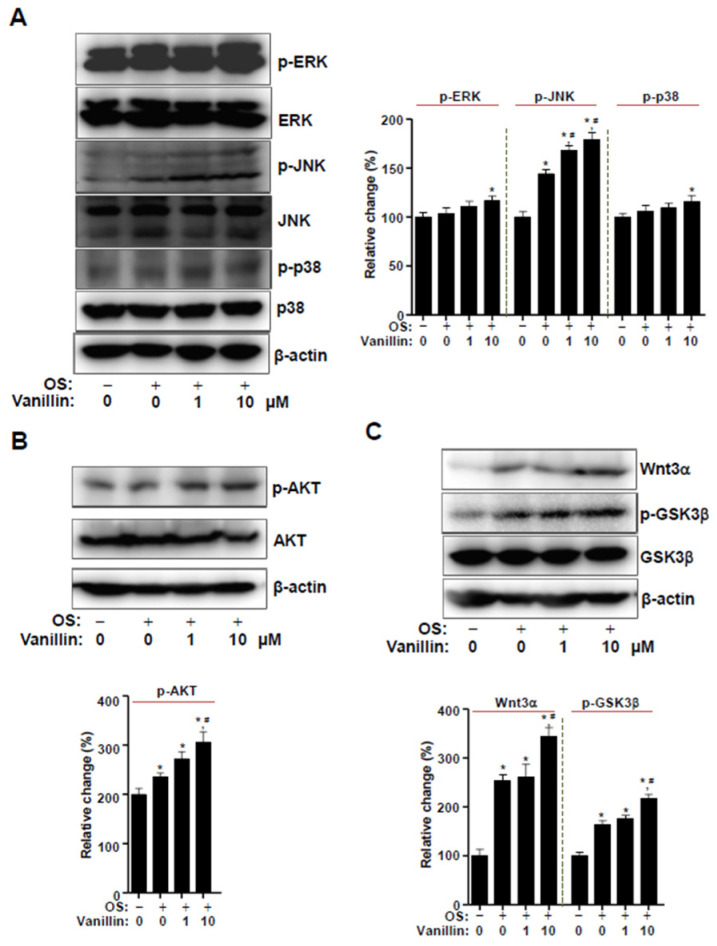
Effect of vanillin on BMP2-related signaling in osteoblasts. (**A**–**C**) Cells were treated in OS with vanillin for 3 days. Western blot analysis was performed to investigate the expression of ERK, p-ERK, JNK, p-JNK, p38, p-p38, and β-actin (**A**); AKT, p-AKT, and β-actin (**B**); Wnt3a, GSK3β, p-GSK3β, and β-actin. The relative change (%) is shown as a bar graph. Data are expressed as the mean ± SD. * *p* < 0.05, versus control. # *p* < 0.05, versus OS. Data represent the results of three experiments.

To further explore the potential molecules closely associated with BMP2 signaling, we placed 1 and 10 μM of vanillin in osteogenic cells and detected the phosphorylation of mitogen-activated protein kinases (MAPKs), the noncanonical pathway of BMP2 signaling, using Western blot analysis. The results revealed that 1 and 10 μM vanillin stimulated JNK phosphorylation compared with the cells incubated with control and OS alone, whereas 10 μM vanillin (but not 1 μM) stimulated ERK1/2 phosphorylation and p38 compared with the cells incubated with control. (Figure 4A). To determine whether vanillin also influences AKT and Wnt3a signaling, we detected AKT phosphorylation, Wnt3a expression, and GSK3β phosphorylation. The results showed that 10 μM vanillin, but not 1 μM, stimulated AKT and Wnt3a signaling compared with the cells incubated with control and OS alone (Figure 4B,C). Thus, these data suggest that the osteogenic-promoting effects of vanillin also involve Wnt3a signaling, which is associated with BMP2 signaling.

### 3.5. Vanillin Enhances F-Actin Polymerization and Migration, and Its Osteogenic Effects Are Attenuated by Blocking BMP2 Signaling

**Figure 5:**
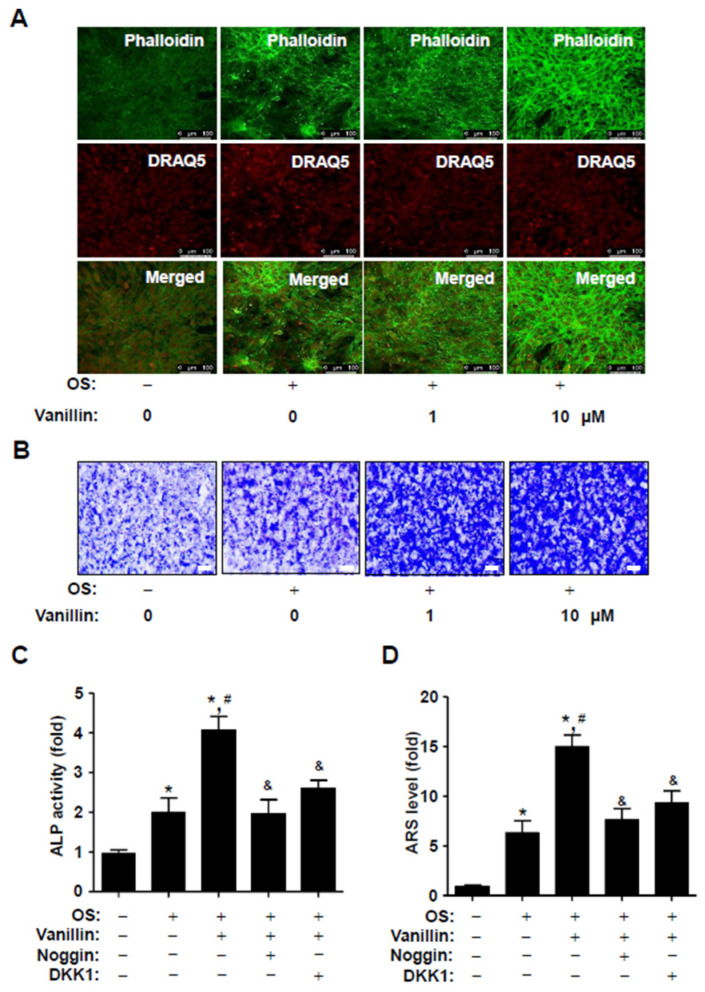
Effects of vanillin on F-actin polymerization, cell migration, and the inhibition of BMP signaling in vanillin-stimulated osteoblast differentiation. (**A**) F-actin polymerization was stained with Fluorescein phalloidin (green), and the cells were stained with DRAQ5 (red). Images shown in the upper and middle panels were observed using multiphoton microscopy, and the images are merged in the lower bottom panels. Scale bar: 50 μm. (**B**) Cell migration was detected using a Matrigel-coated membrane in a Boyden chamber. (**C**,**D**) Vanillin was treated with noggin (10 μg/mL) or DKK1 (0.5 μg/mL) with OS for 7 days (**C**) and 14 days (**D**). Osteoblast differentiation was analyzed by ALP activity (**C**) and ARS staining (**D**). Data are expressed as the mean ± SD. * *p* < 0.05, versus control. # *p* < 0.05, versus OS. & *p* < 0.05, versus vanillin. Data represent the results of three experiments.

Having established that vanillin increases RUNX2 expression via the osteogenic signaling pathway, we investigated whether vanillin also influences morphological phenotypes during osteoblast differentiation. Since BMP2 signaling induces F-actin polymerization, which is increased during osteoblast differentiation and is involved in cell migration, we observed cytoskeletal changes using rhodamine–phalloidin staining in an intravital multiphoton microscope (IMPM). The IMPM observation revealed that vanillin stimulated F-actin polymerization on Matrigel-coated culture plates during osteoblast differentiation (Figure 5A). Subsequently, we monitored the effect on cell migration of vanillin using Boyden chamber assays. The observation revealed that vanillin stimulated migration across the Matrigel-coated membranes (Figure 5B). Finally, we examined the functional consequence of the vanillin-mediated BMP2 and its related signaling on osteoblast differentiation; vanillin was treated in the presence or absence of the BMP2 antagonist, noggin, and Wnt/β-catenin-signaling inhibitor Dickkopf-1 (DKK1). Noggin and DKK1 pretreatment statistically attenuated vanillin-stimulated ALP activity and the ARS level during early and late differentiation (Figure 5C,D). Thus, these data suggest that vanillin stimulates osteogenic processes through BMP2 signaling in osteogenic cells.

### 3.6. Vanillin Enhances Cell Survival through Antioxidant Effects in Osteoblast Differentiation

**Figure 6:**
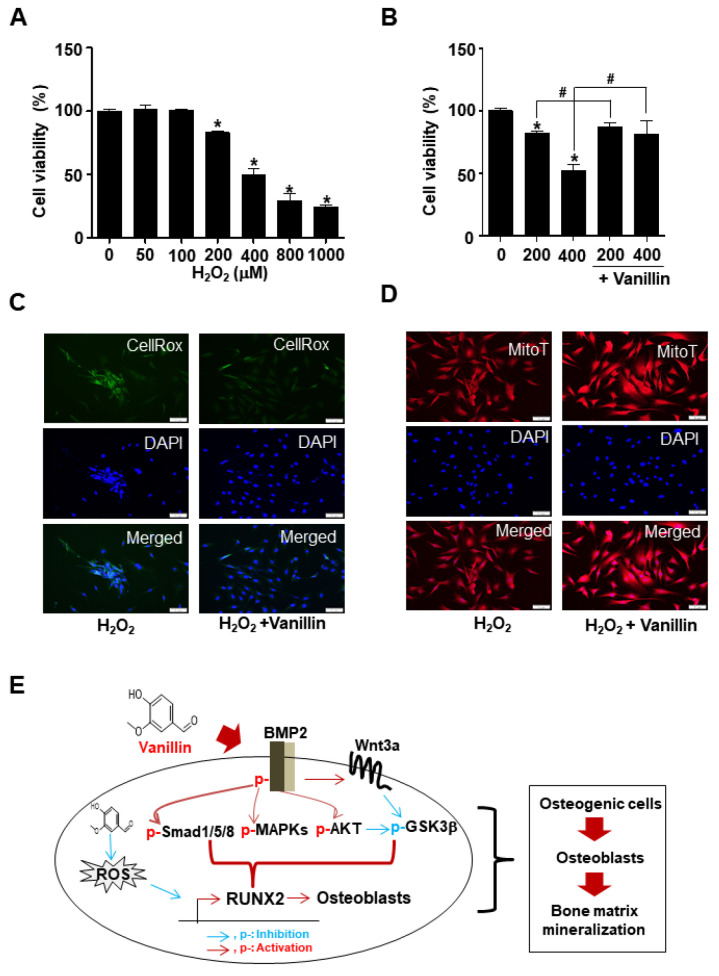
Antioxidant effects on oxidative stress of vanillin in osteoblasts. (**A**) Cells were treated in OS with indicated concentration of H_2_O_2_, and cell viability was determined using MTT assay. (**B**) Oxidative stress-induced cells in OS were treated with vanillin, and cell viability was determined using MTT assay. (**C**,**D**) ROS level (**C**) and active mitochondria (**D**) in oxidative stress-induced cells were detected using CellROX™ Green reagent and MitoTracker™ Red CMXRos, respectively. Scale bar: 50 μm. (**E**) Proposed model underlying antioxidant vanillin as protective compound in osteoblast differentiation and survival. Data are expressed as the mean ± SD. * *p* < 0.05, versus control. # *p* < 0.05, versus vanillin. Data represent the results of three experiments.

Reactive oxygen species (ROS) are a main source of osteoblast cell death. We finally investigated whether the antioxidant activity of vanillin influences osteoblast cell death. We treated 0–1000 μM H_2_O_2_ and analyzed the subsequent cell viability using an MTT assay. The results indicated that H_2_O_2_ induces cell death, starting at a concentration of 200 μM (50 μM: *p* value = 0.6360; 100 μM: *p* value = 0.8490; 200 μM: *p* value = 0.0008; 400 μM: *p* value = 0.0006; 800 μM: *p* value = 0.0003; 1000 μM: *p* value < 0.0001) (Figure 6A). Vanillin was treated in the presence or absence of 200 μM or 400 μM H_2_O_2_ during osteoblast differentiation, and the MTT results revealed that vanillin prevented cell death caused by oxidative stress (Figure 6B). To further validate the effect of vanillin on oxidative stress, CellROX™ Green reagent was used to measure the ROS level, and MitoTracker™ Red CMXRos was used to measure active mitochondria. Vanillin reduced the ROS accumulation and increased the mitochondria stain level (Figure 6C,D). Thus, these data suggest that vanillin prevents osteoblast damage and cell death caused by oxidative stress.

## 4. Discussion

Osteoblast differentiation and survival is an essential process of bone formation. By stimulating osteoblast differentiation, anabolic medicines derived from natural compounds can be utilized to treat and prevent bone diseases [23,24,25,26]. Recently, we reported the beneficial effects of various natural compounds isolated from plants on osteogenic cells [3,26,27,28,29,30,31]. For osteoblast differentiation in in vitro research, native bone marrow-derived osteoblasts and other cell sources were studied [32,33]. However, the osteogenic MC3T3-E1 cells derived from calvaria are commonly used for osteoblast differentiation in in vitro research due to their relatively pure population. In the present study, using calvaria-derived osteogenic MC3T3-E1 cells as an in vitro cell system, we demonstrated for the first time that antioxidant vanillin purified from *Adenophora triphylla var. japonica* showed osteogenic-promoting effects to induce osteoblast differentiation and survival, suggesting vanillin as a bone-protective compound.

Bone formation and repair are complex processes including osteoblast differentiation, matrix mineralization, and osteoblast survival [34,35,36,37,38]. Osteoblast differentiation causes bone-forming activities and matrix mineralization, whereas impaired osteoblast regulation and cell death causes bone diseases such as osteoporosis, periodontitis, and osteonecrosis [39,40,41,42]. In the present study, we first demonstrated the cytotoxicity of vanillin in osteogenic cells using an MTT assay. We found that vanillin did not induce cytotoxicity in the MTT assay. Under this condition, we demonstrated that vanillin stimulates ALP enzymatic activity. ALP activity is a key marker for early osteoblast differentiation, and the ARS staining level is a phenotypic marker for final differentiation, osteoblast maturation, and calcium deposition in matrix mineralization [31,43]. Subsequently, crystals of calcium and phosphate, known as hydroxyapatite, are embedded on the extracellular matrix for bone tissue mineralization [44,45]. Our results also validated that vanillin increases the mineralized matrix formation through terminal osteoblast differentiation. These data suggest that early and terminal osteoblast differentiation and mineralization are promoted by vanillin.

BMP2 signaling is triggered by interactions with its type I receptor (BMPRI) and type II receptor (BMPRII). In the present study, our data demonstrated that vanillin enhanced BMP2 expression, Smad1/5/8 phosphorylation, MAPK phosphorylation, and RUNX2 expression. BMP2 signaling is also associated with Wnt3a signaling and consequently controls RUNX2 expression [46,47,48,49]. Upon activation, its receptors induce the phosphorylation of the downstream proteins, Smad 1/5/8 and MAPK. The activated Smad 1/5/8 complexes with Smad4; the complex is translocated to the nucleus; and the complex and MAPKs finally regulate RUNX2 activity and expression [50,51,52,53]. In addition, BMP2 and Wnt3a activates AKT serine–threonine kinase activity, and AKT signaling also enhances osteoblast differentiation [54,55,56]. We also found that vanillin increases Wnt3a and AKT signaling. We also demonstrated that vanillin-mediated ALP activity and matrix mineralization are attenuated by pharmacological inhibition, using BMP2 and Wnt3a antagonists. These findings suggest that vanillin stimulates BMP2 signaling, and its associated pathways are required for osteoblast differentiation and maturation in osteogenic cells

Actin is one of the three major components of the cytoskeleton, which is distributed within cells in the form of G-actin or F-actin [57,58]. Our present study showed that vanillin increases F-actin polymerization during osteoblast differentiation. F-actin is a linear polymer microfilament composed of G-actin monomers. F-actin is known to be involved in cell division, cell migration, and cell invasion, and it was recently shown that F-actin regulates cell fate and cell differentiation [59,60,61,62,63]. F-actin depolymerization increases adipogenic differentiation, while F-actin polymerization enhances osteoblast differentiation and bone formation [58,60,63,64,65]. Osteoblast migration has a functional role in bone development, bone formation, and bone fracture repair [41,66,67,68,69,70,71]. In the present study, we demonstrated that vanillin accelerates the migration of osteoblasts into the extracellular matrix. BMP2 signaling induces actin cytoskeleton reorganization and is also involved in the migration of cells such as bone marrow mesenchymal progenitors, osteoblasts, and endothelial cells [66,67,72,73,74]. Based on our findings, it is consistent with the vanillin-stimulated early and terminal osteoblast differentiation through BMP2 signaling. These findings suggest that vanillin has an anabolic effect, to accelerate osteogenic processes through F-actin polymerization and migration in osteogenic cells.

As a powerful antioxidant, vanillin shows cellular protective effects against oxidative stress [18,75,76,77,78,79,80]. Vanillin has a stronger antioxidant activity than ascorbic acid, and the authors suggested that the antioxidant activity of vanillin is beneficial for daily health care [18]. In the present study, we demonstrated that vanillin prevents ROS-induced cell death. Vanillin suppresses hepatic lipid peroxidation and inhibits the carbon tetrachloride-mediated depletion of the antioxidant enzyme and glutathione level in the liver [79]. Similar with the previous literature, our present results demonstrated that vanillin prevents oxidative stress and mitochondria damage in osteoblasts. Thus, our findings suggest that, due to its antioxidant activity, vanillin increases osteoblast survival in bone tissue by preventing oxidative damage, thereby increasing bone formation.

## 5. Conclusions

In conclusion, the present study is the first report that vanillin purified from *A. triphylla var. japonica* stimulates osteoblast differentiation, maturation, and survival by increasing BMP2 signaling, RUNX2 expression, F-actin polymerization, migration, and antioxidant capacity, resulting in bone matrix calcification in an in vitro cell system (Figure 6E). In future studies, reverification using primary cultured osteoblast cells, other cell sources, and in vivo animal experiments should be explored to further confirm vanillin-induced bone formation and protective effects against skeletal disorders. Despite the limitations of the present study, our in vitro results suggest vanillin as a new compound regulating osteogenic processes, and they provide future perspectives for the use of vanillin as a bone-protective compound in daily health supplements or for skeletal disorders such as osteoporosis.

## Data Availability

The data generated during the current study are available from the corresponding author on reasonable request.

## References

[1] Fakhry M., Hamade E., Badran B., Buchet R., Magne D. (2013). Molecular mechanisms of mesenchymal stem cell differentiation towards osteoblasts. World J. Stem Cells.

[2] Phan T.C., Xu J., Zheng M.H. (2004). Interaction between osteoblast and osteoclast: Impact in bone disease. Histol. Histopathol..

[3] Park K.R., Lee J.Y., Kim B.M., Kang S.W., Yun H.M. (2020). TMARg, a Novel Anthraquinone Isolated from Rubia cordifolia Nakai, Increases Osteogenesis and Mineralization through BMP2 and beta-Catenin Signaling. Int. J. Mol. Sci..

[4] Lee H.R., Kim H.M., Jeong H.W., Kim G.G., Na C.I., Oh M.M., Hwang S.J. (2019). Growth Characteristics of Adenophora triphylla var. japonica Hara Seedlings as Affected by Growing Medium. Plants.

[5] Mlynarczyk K., Walkowiak-Tomczak D., Lysiak G.P. (2018). Bioactive properties of Sambucus nigra L. as a functional ingredient for food and pharmaceutical industry. J. Funct. Foods.

[6] Konno C., Saito T., Oshima Y., Hikino H., Kabuto C. (1981). Structure of methyl adenophorate and triphyllol, triterpenoids of Adenophora triphylla var. japonica roots. Planta Med..

[7] Hu J.R., Jung C.J., Ku S.M., Jung D.H., Ku S.K., Choi J.S. (2019). Antitussive, expectorant, and anti-inflammatory effects of Adenophorae Radix powder in ICR mice. J. Ethnopharmacol..

[8] Lee D.R., Lee Y.S., Choi B.K., Lee H.J., Park S.B., Kim T.M., Oh H.J., Yang S.H., Suh J.W. (2015). Roots extracts of Adenophora triphylla var. japonica improve obesity in 3T3-L1 adipocytes and high-fat diet-induced obese mice. Asian Pac. J. Trop. Med..

[9] Chun J., Kang M., Kim Y.S. (2014). A triterpenoid saponin from Adenophora triphylla var. japonica suppresses the growth of human gastric cancer cells via regulation of apoptosis and autophagy. Tumor Biol..

[10] Schreck K., Melzig M.F. (2021). Traditionally Used Plants in the Treatment of Diabetes Mellitus: Screening for Uptake Inhibition of Glucose and Fructose in the Caco2-Cell Model. Front. Pharmacol..

[11] Kang M., Ha I.J., Chun J., Kang S.S., Kim Y.S. (2013). Separation of two cytotoxic saponins from the roots of *Adenophora triphylla* var. *japonica* by high-speed counter-current chromatography. Phytochem. Anal..

[12] Kim S.J., Cho H.I., Kim S.J., Kim J.S., Kwak J.H., Lee D.U., Lee S.K., Lee S.M. (2014). Protective effects of lupeol against D-galactosamine and lipopolysaccharide-induced fulminant hepatic failure in mice. J. Nat. Prod..

[13] Ahn E.K., Oh J.S. (2013). Lupenone isolated from Adenophora triphylla var. japonica extract inhibits adipogenic differentiation through the downregulation of PPARgamma in 3T3-L1 cells. Phytother. Res..

[14] Kim D., Kim K.Y. (2021). Adenophora triphylla var. japonica Inhibits Candida Biofilm Formation, Increases Susceptibility to Antifungal Agents and Reduces Infection. Int. J. Mol. Sci..

[15] Zhao D., Jiang Y., Sun J., Li H., Huang M., Sun X., Zhao M. (2019). Elucidation of The Anti-Inflammatory Effect of Vanillin In Lps-Activated THP-1 Cells. J. Food Sci..

[16] Sinha A.K., Sharma U.K., Sharma N. (2008). A comprehensive review on vanilla flavor: Extraction, isolation and quantification of vanillin and others constituents. Int. J. Food Sci. Nutr..

[17] Arya S.S., Rookes J.E., Cahill D.M., Lenka S.K. (2021). Vanillin: A review on the therapeutic prospects of a popular flavouring molecule. Adv. Tradit. Med..

[18] Tai A., Sawano T., Yazama F., Ito H. (2011). Evaluation of antioxidant activity of vanillin by using multiple antioxidant assays. Biochim. Biophys. Acta.

[19] Sawa T., Nakao M., Akaike T., Ono K., Maeda H. (1999). Alkylperoxyl radical-scavenging activity of various flavonoids and other phenolic compounds: Implications for the anti-tumor-promoter effect of vegetables. J. Agric. Food Chem..

[20] Chen Y.Q., Dou C., Yi J., Tang R.H., Yu T., Zhou L., Luo W., Liang M.M., Yin X.L., Li J.M. (2018). Inhibitory effect of vanillin on RANKL-induced osteoclast formation and function through activating mitochondrial-dependent apoptosis signaling pathway. Life Sci..

[21] Yun H.M., Kim B., Park J.E., Park K.R. (2022). Trifloroside Induces Bioactive Effects on Differentiation, Adhesion, Migration, and Mineralization in Pre-Osteoblast MC3T3E-1 Cells. Cells.

[22] Park K.R., Park J.E., Kim B., Kwon I.K., Hong J.T., Yun H.M. (2021). Calycosin-7-O-beta-Glucoside Isolated from Astragalus membranaceus Promotes Osteogenesis and Mineralization in Human Mesenchymal Stem Cells. Int. J. Mol. Sci..

[23] Mishra B.B., Tiwari V.K. (2011). Natural products: An evolving role in future drug discovery. Eur. J. Med. Chem..

[24] An J., Yang H., Zhang Q., Liu C., Zhao J., Zhang L., Chen B. (2016). Natural products for treatment of osteoporosis: The effects and mechanisms on promoting osteoblast-mediated bone formation. Life Sci..

[25] Soelaiman I.N., Das S., Shuid A.N., Mo H., Mohamed N. (2013). Use of medicinal plants and natural products for treatment of osteoporosis and its complications. Evid. Based Complement Altern. Med..

[26] Yun H.M., Lee J.Y., Kim S.H., Kwon I.K., Park K.R. (2022). Effects of Triterpene Soyasapogenol B from Arachis hypogaea (Peanut) on Differentiation, Mineralization, Autophagy, and Necroptosis in Pre-Osteoblasts. Int. J. Mol. Sci..

[27] Yun H.M., Kim B., Jeong Y.H., Hong J.T., Park K.R. (2023). Suffruticosol A elevates osteoblast differentiation targeting BMP2-Smad/1/5/8-RUNX2 in pre-osteoblasts. Biofactors.

[28] Park K.R., Kim B., Lee J.Y., Moon H.J., Kwon I.K., Yun H.M. (2022). Effects of Scoparone on differentiation, adhesion, migration, autophagy and mineralization through the osteogenic signalling pathways. J. Cell. Mol. Med..

[29] Park K.R., Lee J.Y., Cho M., Yun H.M. (2021). Ziyuglycoside I Upregulates RUNX2 through ERK1/2 in Promoting Osteoblast Differentiation and Bone Mineralization. Am. J. Chin. Med..

[30] Park K.R., Leem H.H., Cho M., Kang S.W., Yun H.M. (2020). Effects of the amide alkaloid piperyline on apoptosis, autophagy, and differentiation of pre-osteoblasts. Phytomedicine.

[31] Yun H.M., Park K.R., Quang T.H., Oh H., Hong J.T., Kim Y.C., Kim E.C. (2015). 2,4,5-Trimethoxyldalbergiquinol promotes osteoblastic differentiation and mineralization via the BMP and Wnt/beta-catenin pathway. Cell Death Dis..

[32] Scala R., Maqoud F., Angelelli M., Latorre R., Perrone M.G., Scilimati A., Tricarico D. (2019). Zoledronic Acid Modulation of TRPV1 Channel Currents in Osteoblast Cell Line and Native Rat and Mouse Bone Marrow-Derived Osteoblasts: Cell Proliferation and Mineralization Effect. Cancers.

[33] Savino S., Toscano A., Purgatorio R., Profilo E., Laghezza A., Tortorella P., Angelelli M., Cellamare S., Scala R., Tricarico D. (2018). Novel bisphosphonates with antiresorptive effect in bone mineralization and osteoclastogenesis. Eur. J. Med. Chem..

[34] Histing T., Stenger D., Kuntz S., Scheuer C., Tami A., Garcia P., Holstein J.H., Klein M., Pohlemann T., Menger M.D. (2012). Increased osteoblast and osteoclast activity in female senescence-accelerated, osteoporotic SAMP6 mice during fracture healing. J. Surg. Res..

[35] Zayzafoon M. (2006). Calcium/calmodulin signaling controls osteoblast growth and differentiation. J. Cell. Biochem..

[36] Broz A., Ukraintsev E., Kromka A., Rezek B., Hubalek Kalbacova M. (2017). Osteoblast adhesion, migration, and proliferation variations on chemically patterned nanocrystalline diamond films evaluated by live-cell imaging. J. Biomed. Mater. Res. A.

[37] Tome M., Lopez-Romero P., Albo C., Sepulveda J.C., Fernandez-Gutierrez B., Dopazo A., Bernad A., Gonzalez M.A. (2011). miR-335 orchestrates cell proliferation, migration and differentiation in human mesenchymal stem cells. Cell Death Differ..

[38] Yang Y., Zhang T., Jiang M., Yin X., Luo X., Sun H. (2021). Effect of the immune responses induced by implants in a integrated three-dimensional micro-nano topography on osseointegration. J. Biomed. Mater. Res. A.

[39] Deng T., Zhang W., Zhang Y., Zhang M., Huan Z., Yu C., Zhang X., Wang Y., Xu J. (2021). Thyroid-stimulating hormone decreases the risk of osteoporosis by regulating osteoblast proliferation and differentiation. BMC Endocr. Disord..

[40] Shalehin N., Hosoya A., Takebe H., Hasan M.R., Irie K. (2020). Boric acid inhibits alveolar bone loss in rat experimental periodontitis through diminished bone resorption and enhanced osteoblast formation. J. Dent. Sci..

[41] Infante A., Rodriguez C.I. (2018). Osteogenesis and aging: Lessons from mesenchymal stem cells. Stem Cell Res. Ther..

[42] Karsenty G., Wagner E.F. (2002). Reaching a genetic and molecular understanding of skeletal development. Dev. Cell.

[43] Lee H.S., Jung E.Y., Bae S.H., Kwon K.H., Kim J.M., Suh H.J. (2011). Stimulation of osteoblastic differentiation and mineralization in MC3T3-E1 cells by yeast hydrolysate. Phytother. Res..

[44] Lin X., Patil S., Gao Y.G., Qian A. (2020). The Bone Extracellular Matrix in Bone Formation and Regeneration. Front. Pharmacol..

[45] Ogata Y. (2008). Bone sialoprotein and its transcriptional regulatory mechanism. J. Periodontal Res..

[46] Rawadi G., Vayssiere B., Dunn F., Baron R., Roman-Roman S. (2003). BMP-2 controls alkaline phosphatase expression and osteoblast mineralization by a Wnt autocrine loop. J. Bone Miner. Res..

[47] Gaur T., Lengner C.J., Hovhannisyan H., Bhat R.A., Bodine P.V., Komm B.S., Javed A., van Wijnen A.J., Stein J.L., Stein G.S. (2005). Canonical WNT signaling promotes osteogenesis by directly stimulating Runx2 gene expression. J. Biol. Chem..

[48] Salazar V.S., Ohte S., Capelo L.P., Gamer L., Rosen V. (2016). Specification of osteoblast cell fate by canonical Wnt signaling requires Bmp2. Development.

[49] Fujita K., Janz S. (2007). Attenuation of WNT signaling by DKK-1 and -2 regulates BMP2-induced osteoblast differentiation and expression of OPG, RANKL and M-CSF. Mol. Cancer.

[50] Canalis E., Economides A.N., Gazzerro E. (2003). Bone morphogenetic proteins, their antagonists, and the skeleton. Endocr. Rev..

[51] Harris S.E., Guo D., Harris M.A., Krishnaswamy A., Lichtler A. (2003). Transcriptional regulation of BMP-2 activated genes in osteoblasts using gene expression microarray analysis: Role of Dlx2 and Dlx5 transcription factors. Front. Biosci..

[52] Javed A., Afzal F., Bae J.S., Gutierrez S., Zaidi K., Pratap J., van Wijnen A.J., Stein J.L., Stein G.S., Lian J.B. (2009). Specific residues of RUNX2 are obligatory for formation of BMP2-induced RUNX2-SMAD complex to promote osteoblast differentiation. Cells Tissues Organs.

[53] Bae J.S., Gutierrez S., Narla R., Pratap J., Devados R., van Wijnen A.J., Stein J.L., Stein G.S., Lian J.B., Javed A. (2007). Reconstitution of Runx2/Cbfa1-null cells identifies a requirement for BMP2 signaling through a Runx2 functional domain during osteoblast differentiation. J. Cell. Biochem..

[54] Ghosh-Choudhury N., Abboud S.L., Nishimura R., Celeste A., Mahimainathan L., Choudhury G.G. (2002). Requirement of BMP-2-induced phosphatidylinositol 3-kinase and Akt serine/threonine kinase in osteoblast differentiation and Smad-dependent BMP-2 gene transcription. J. Biol. Chem..

[55] Dong J., Xu X., Zhang Q., Yuan Z., Tan B. (2020). The PI3K/AKT pathway promotes fracture healing through its crosstalk with Wnt/beta-catenin. Exp. Cell. Res..

[56] Fang Y., Xue Z., Zhao L., Yang X., Yang Y., Zhou X., Feng S., Chen K. (2019). Calycosin stimulates the osteogenic differentiation of rat calvarial osteoblasts by activating the IGF1R/PI3K/Akt signaling pathway. Cell Biol. Int..

[57] Ono S. (2007). Mechanism of depolymerization and severing of actin filaments and its significance in cytoskeletal dynamics. Int. Rev. Cytol..

[58] Khan A.U., Qu R., Fan T., Ouyang J., Dai J. (2020). A glance on the role of actin in osteogenic and adipogenic differentiation of mesenchymal stem cells. Stem Cell Res. Ther..

[59] Han Y., Kim S.J. (2018). Simvastatin-dependent actin cytoskeleton rearrangement regulates differentiation via the extracellular signal-regulated kinase-1/2 and p38 kinase pathways in rabbit articular chondrocytes. Eur. J. Pharmacol..

[60] Chen L., Hu H., Qiu W., Shi K., Kassem M. (2018). Actin depolymerization enhances adipogenic differentiation in human stromal stem cells. Stem Cell Res..

[61] Akhshi T.K., Wernike D., Piekny A. (2014). Microtubules and actin crosstalk in cell migration and division. Cytoskeleton.

[62] Meirson T., Gil-Henn H. (2018). Targeting invadopodia for blocking breast cancer metastasis. Drug Resist. Updates.

[63] Tong Z., Liu Y., Xia R., Chang Y., Hu Y., Liu P., Zhai Z., Zhang J., Li H. (2020). F-actin Regulates Osteoblastic Differentiation of Mesenchymal Stem Cells on TiO(2) Nanotubes Through MKL1 and YAP/TAZ. Nanoscale Res. Lett..

[64] Chen L., Shi K., Frary C.E., Ditzel N., Hu H., Qiu W., Kassem M. (2015). Inhibiting actin depolymerization enhances osteoblast differentiation and bone formation in human stromal stem cells. Stem Cell Res..

[65] Xue X., Hong X., Li Z., Deng C.X., Fu J. (2017). Acoustic tweezing cytometry enhances osteogenesis of human mesenchymal stem cells through cytoskeletal contractility and YAP activation. Biomaterials.

[66] Fiedler J., Roderer G., Gunther K.P., Brenner R.E. (2002). BMP-2, BMP-4, and PDGF-bb stimulate chemotactic migration of primary human mesenchymal progenitor cells. J. Cell. Biochem..

[67] Lind M., Eriksen E.F., Bunger C. (1996). Bone morphogenetic protein-2 but not bone morphogenetic protein-4 and -6 stimulates chemotactic migration of human osteoblasts, human marrow osteoblasts, and U2-OS cells. Bone.

[68] Granero-Molto F., Weis J.A., Miga M.I., Landis B., Myers T.J., O’Rear L., Longobardi L., Jansen E.D., Mortlock D.P., Spagnoli A. (2009). Regenerative effects of transplanted mesenchymal stem cells in fracture healing. Stem Cells.

[69] Delaisse J.M. (2014). The reversal phase of the bone-remodeling cycle: Cellular prerequisites for coupling resorption and formation. Bonekey Rep..

[70] Kalbacova M., Broz A., Kong J., Kalbac M. (2010). Graphene substrates promote adherence of human osteoblasts and mesenchymal stromal cells. Carbon.

[71] Aryaei A., Jayatissa A.H., Jayasuriya A.C. (2014). The effect of graphene substrate on osteoblast cell adhesion and proliferation. J. Biomed. Mater. Res. Part A.

[72] Gamell C., Osses N., Bartrons R., Ruckle T., Camps M., Rosa J.L., Ventura F. (2008). BMP2 induction of actin cytoskeleton reorganization and cell migration requires PI3-kinase and Cdc42 activity. J. Cell Sci..

[73] Dudas M., Sridurongrit S., Nagy A., Okazaki K., Kaartinen V. (2004). Craniofacial defects in mice lacking BMP type I receptor Alk2 in neural crest cells. Mech. Dev..

[74] Sotobori T., Ueda T., Myoui A., Yoshioka K., Nakasaki M., Yoshikawa H., Itoh K. (2006). Bone morphogenetic protein-2 promotes the haptotactic migration of murine osteoblastic and osteosarcoma cells by enhancing incorporation of integrin beta1 into lipid rafts. Exp. Cell Res..

[75] Zhu J., Chen X., Huang T., Tian D., Gao R. (2023). Characterization and antioxidant properties of chitosan/ethyl-vanillin edible films produced via Schiff-base reaction. Food Sci. Biotechnol..

[76] Liu Y.N., Kang J.W., Zhang Y., Song S.S., Xu Q.X., Zhang H., Lu L., Wei S.W., Liang C., Su R.W. (2023). Vanillin prevents the growth of endometriotic lesions through anti-inflammatory and antioxidant pathways in a mouse model. Food Funct..

[77] Xiong S., Li R., Ye S., Ni P., Shan J., Yuan T., Liang J., Fan Y., Zhang X. (2022). Vanillin enhances the antibacterial and antioxidant properties of polyvinyl alcohol-chitosan hydrogel dressings. Int. J. Biol. Macromol..

[78] Salau V.F., Erukainure O.L., Ibeji C.U., Olasehinde T.A., Koorbanally N.A., Islam M.S. (2020). Vanillin and vanillic acid modulate antioxidant defense system via amelioration of metabolic complications linked to Fe^2+^-induced brain tissues damage. Metab. Brain Dis..

[79] Makni M., Chtourou Y., Fetoui H., Garoui E.M., Boudawara T., Zeghal N. (2011). Evaluation of the antioxidant, anti-inflammatory and hepatoprotective properties of vanillin in carbon tetrachloride-treated rats. Eur. J. Pharmacol..

[80] Kamat J.P., Ghosh A., Devasagayam T.P. (2000). Vanillin as an antioxidant in rat liver mitochondria: Inhibition of protein oxidation and lipid peroxidation induced by photosensitization. Mol. Cell. Biochem..

